# Tracking a single pigeon using a shadowing filter algorithm

**DOI:** 10.1002/ece3.2976

**Published:** 2017-05-11

**Authors:** Ayham Zaitouny, Thomas Stemler, Michael Small

**Affiliations:** ^1^ School of Mathematics and StatisticsUniversity of Western AustraliaCrawleyWAAustralia; ^2^ Commonwealth Scientific and Industrial Research Organisation (CSIRO)KensingtonWAAustralia; ^3^ Potsdam Institute for Climate Impact Research (PIK)PotsdamAustralia

**Keywords:** animal behavior, animal movement, filtering, GPS, shadowing filter, tracking

## Abstract

Miniature GPS devices now allow for measurement of the movement of animals in real time and provide high‐ quality and high‐resolution data. While these new data sets are a great improvement, one still encounters some measurement errors as well as device failures. Moreover, these devices only measure position and require further reconstruction techniques to extract the full dynamical state space with the velocity and acceleration. Direct differentiation of position is generally not adequate. We report on the successful implementation of a shadowing filter algorithm that (1) minimizes measurement errors and (2) reconstructs at the same time the full phase‐space from a position recording of a flying pigeon. This filter is based on a very simple assumption that the pigeon's dynamics are Newtonian. We explore not only how to choose the filter's parameters but also demonstrate its improvements over other techniques and give minimum data requirements. In contrast to competing filters, the shadowing filter's approach has not been widely implemented for practical problems. This article addresses these practicalities and provides a prototype for such application.

## 1 Introduction

One of the most conceptual and challenging problems in animal behavior is understanding how animals move within a group or flock. In other words, what we call “animal collective behavior” (Sumpter, 2010). In fact, understanding the principles of animal behavior has numerous benefits to mankind. To name a few, studying animal collective behavior allows (1) design of optimal algorithms simulation and forecasting of animal dynamics which are significantly important for farms and wildlife sanctuaries management and (2) develops a coherent engineering machine dynamics of groups of microrobots.

Despite the importance of understanding animal movement, it has only been during the last two decades that researchers and scientists have been able to directly study and understand animal collective motion and behavior (Sumpter, 2010). The difficulty has been a lack of real data of sufficient precision and frequency with which to verify models. However, in the last few years, particularly because of new technology such as Global Positing System (GPS) devices and video recording systems, the interest in studying animal motion and collective behavior in vertebrates has increased—both from biology (Godley, Broderick, Glen & Hays, 2003; Ryan, Petersen, Peters & Gremillet, 2004) and from physics (Kattas, Xu & Small, 2012; Kattas, Perez‐Barberia, Small, Xu & Walker, 2013). In particular, this new technology allows researchers to record large spatial data sets of animal motion, which then opens the door for better validated models and better understanding of collective and individual animal dynamics (Bonabeau, Dorigo & Theraulaz, 1999).

While these new data sets are a substantial improvement, we are still faced with significant challenges due to measurement errors and device failures. Moreover, these devices only record information about position, while exploring animal behavior in detail requires the full phase‐state, including velocity and acceleration. Therefore, before using a such raw motion data for further investigations, it is important to clean and analyze the data properly. In this study, we introduce a new versatile tracking methodology to overcome all these challenges. Specifically, the focus of our article is GPS data tracking pigeons’ behavior. We look at the problem of how best to interpret and filter the raw data from an avian‐mounted GPS transponder (the individual pigeons carry a small GPS “backpack”) to provide a meaningful flight trajectory. The GPS data are provided by (Nagy, Akos, Biro & Vicsek, 2010). In their study, they explored the leadership relation in the flock, they used the same GPS data; however, they cleaned data set and interpolated the missing points using a statistical filter, and they then estimated the velocity and acceleration using direct differentiation.^1^ Consequently, the data include some unusual or unrealistic measurements.

Our main purpose is to understand the flocking phenomena of pigeons (Zaitouny, Stemler & Small, 2017). However, before we are able to examine the collective behavior of the flock, it is important to analyze the raw GPS data from each pigeon. The fundamental problem we wish to address is how well we can rely on this data set for further investigations. Standard filtering methods do exist; however, in this article, we will argue that the shadowing filter is the correct approach and provides superior results to these standard methods. In what follows we implement a shadowing filter to verify the reliability of the data set. The reasons behind choosing shadowing filters are that they follow a very simple but powerful paradigm; that is, if the model we construct is a good one, then the estimations must be consistent with the observations. Shadowing filters approach problems from the point of view of dynamical systems—they have been shown to provide better results when dealing with incomplete information and nonlinear problems than Kalman or Particle filters (Judd, Reynolds & Rosmond, 2004; Judd & Stemler, 2009; Stemler & Judd, 2009). Therefore, the motion‐tracking problem we are faced with is particularly well suited to the shadowing filter approach.

In this article, for potential users, we provide a guide to employing the proposed tracking methodology. Consequently, it is implemented and applied to real data sets of flying pigeons in order to investigate the capability and applicability of the method for such applications on animal movement as well as to investigate the reliability of the GPS data—certainly, we expect errors in these raw data. Moreover, our tracking technique applied to these data provides a robust and direct estimate of the corresponding acceleration within the trajectory. While our particular interest is in the flocking dynamics of pigeons, these methods are, of course, generic and equally well suited to a wide range of biological tracking problems where GPS data are now routinely collected (Steiner et al., 2000; Godley et al., 2003; Gremillet, Dell'Omo, Ryan, Peters, Ropert‐Coudert & Weeks, 2004; Ryan et al., 2004; DeCesare, Squires & Kolbe, 2005; Kattas et al., 2012, 2013).

## 2 Materials and Methods

### 2.1 Data overview: Pigeon data analysis

The data we are using is provided by the authors of (Nagy et al., 2010). The data provide preprocessed and high‐ resolutions trajectories of pigeons flying in a flock. The pigeon's roost is in Budapest, north of the city center on the island of Obudaiziget in the Danube. These data have been obtained from original location observations provided by miniature GPS devices carried by each pigeon in the flock. The GPS devices were designed to log data points of latitude, longitude and altitude coordinates with a time resolution of 0.2 s.

The flight trajectories were smoothed and filtered by Gaussian filter with $\sigma^{2}\;=\;0.4s^{2}$. In case of missing data points due to failure of the GPS devices, the missing positions were interpolated by averaging the before and after recorded data points, and the cubic B‐spline method was used to fit curves onto the points obtained with the 0.2 s sampling rate. The GPS signal was provided for each pigeon, where signal 1 refers to a data point measured by the device, while signal 0 refers to an interpolated data point. We are working only from the data obtained after this filtering.

The data files include two different patterns of flight: free flights and homing flights. In each case, the flocking pigeons were labeled by letters from A to M (the identifiers are unique and fixed between data sets). Despite the GPS devices only measuring position, the recorded data file of each single pigeon also includes data of the velocity and the acceleration, which have been estimated using methods described in (Nagy et al., 2010).

The behavior of all pigeons is similar, but pigeon A is the most active bird and therefore gives us the longest flight trajectories. Consequently, we focus on this data set as our primary example throughout the text of this article.

#### 2.1.1 Free flight

**Figure 1:**
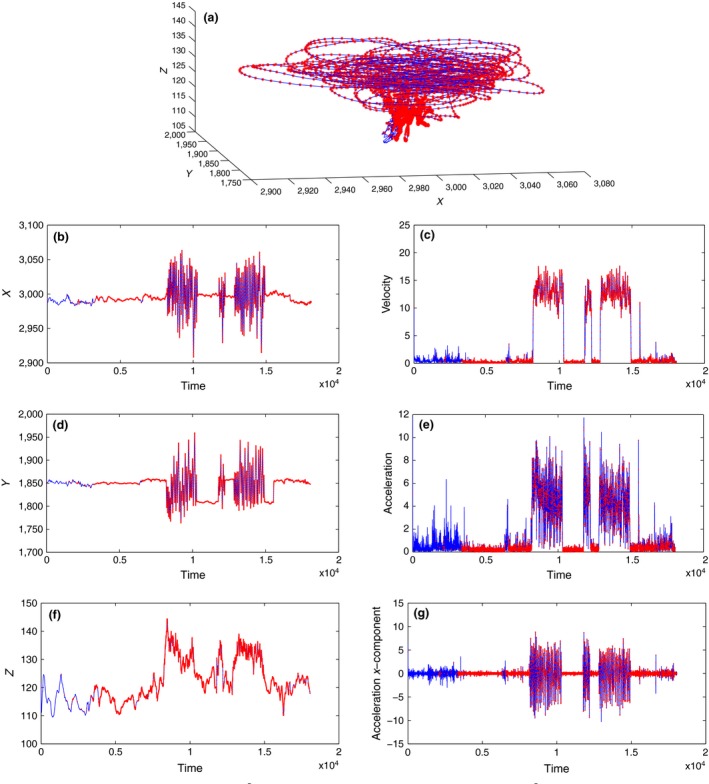
Free Flight: Pigeon A position, velocity, and acceleration. Blue shows the whole trajectory, while red indicates the interpolated data points not logged by the GPS device. (a) Whole trajectory. (b) *X* coordinates. (c) Velocity magnitude. (d) *Y* coordinates. (e) Acceleration magnitude. (f) *Z* coordinates. (g) *X* coordinates acceleration

Figure 1 shows a single pigeon trajectory (pigeon A) from a free flight file. The red stars refers to interpolated data points. The length of the trajectory is 18,061 points with 6,568 interpolated points due to the failure of the GPS; that is, almost 36% of the trajectory points are not measured and must be interpolated. Figure 1a shows the general behavior of a pigeon in free flight, as it flies along a circular trajectory. Obviously from Figure 1b,d we notice that the data include two different patterns of behavior: flight and nonflight; that is, when the pigeon is flying, large and fast changes occur on *x* and *y* coordinates. Otherwise, when the changes on *x* and *y* are small, the pigeon is not airborne and we can see the noise of the GPS devices. Figure 1f does not exhibit this feature to the same extent in the *z* coordinates. Additionally, if we compare the range of *x* and *y* components with *z* component, which is shown in Table 1, we conclude that when a pigeon is flying, it flies approximately in the *xy*‐plane.

**Table 1:** Free flight: Ranges of *x*,* y* and *z* components of position, velocity and acceleration

Position (m)	*x*	*y*	*z*
Range	2,920→3,060	1,760→1,940	110→145
Velocity m/s	$\dot{x}$	$\dot{y}$	$\dot{z}$
Range	−15→15	−15→15	−2→2
Acceleration m/s${}^{2}$	$\ddot{x}$	$\ddot{y}$	$\ddot{z}$
Range	−8→8	−8→8	−2→2

Figure 1c presents the estimated velocity of pigeon A. Again it shows the two different states (flying or nonflying). Moreover, Table 1 confirms our previous assertion that the pigeon flies approximately in *xy*‐plane. That is, the variation of the *x* and *y* velocity components is almost 30m/s, while in the *z* direction, it is only 4m/s. For our latter investigations, the criterion that we will use to distinguish between these two different behaviors is the velocity magnitude (see Figure 1c); that is, we consider a pigeon flying if this magnitude exceeds 4m/s.

Acceleration is the component that controls the dynamics. Therefore, to understand and explore the behavior of a pigeon in a flock, we have to understand its acceleration. As mentioned previously, the data provide an estimation of acceleration – an estimation conducted by (Nagy et al., 2010) using a Gaussian kernel smoothing algorithm to approximate the acceleration from the position data of the GPS device. Figure 1e,g presents these estimates of the magnitude of acceleration and the *x* component of acceleration, respectively. Again, we see two states (flying and nonflying) and that the flying dynamics can be considered essentially on *xy*‐plane (compare the ranges of the acceleration's components from Table 1).

In the next section, we will introduce our tracking methodology to estimate the instantaneous position using shadowing filters. This method will enable us to compute corresponding velocities and accelerations based on the estimated positions. Then, we will present a comparison between the provided data accelerations and our estimations. Thus, we will be able to check the reliability of the data.

#### 2.1.2 Homing flight

**Figure 2:**
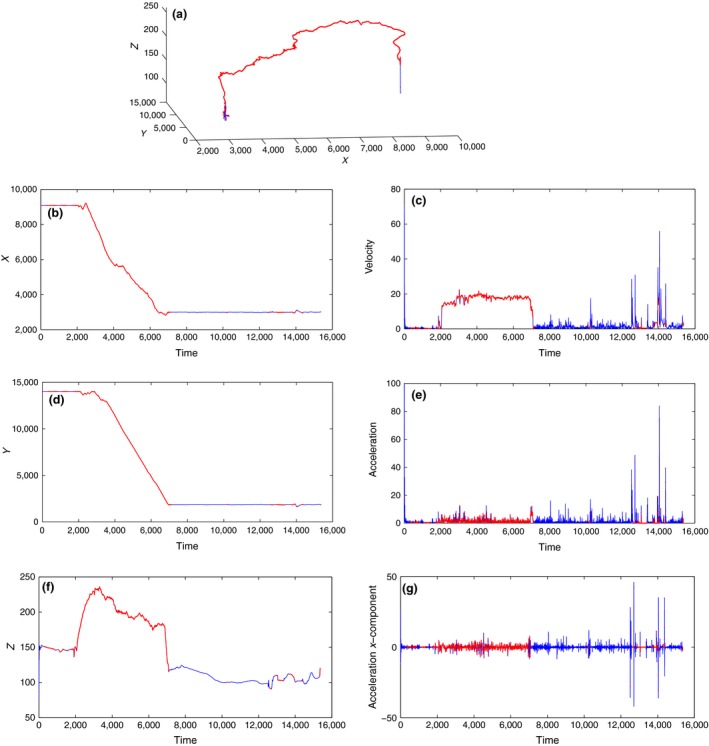
Homing Flight: Pigeon A position, velocity, and acceleration Blue shows the whole trajectory, while red indicates the interpolated data points not logged by the GPS device. (a) Whole trajectory. (b) *X* coordinates. (c) Velocity magnitude. (d) *Y* coordinates. (e) Acceleration magnitude. (f) *Z* coordinates. (g) *X* coordinates acceleration

In this subsection, we analyze homing flight data of pigeon A. In Figure 2, the red stars illustrates the interpolated data points when the GPS device failed to log data. Again, the ratio of non‐GPS to GPS data points is approximately 1:3.

Figure 2a shows that the trajectory of a homing pigeon is almost a linear trajectory, unlike the circular trajectory of a free flight pigeon. Figure 2b,d,f which represents the changes along *x*,*y*, and *z* coordinates, respectively, shows that there are two distinct behaviors as before (flying and nonflying). Moreover, from Table 2, we observe that the range of change along *x* coordinates during the flying period varies from approximately 2,900 to 9,100 m, and along *y* coordinates from 2,000 to 1,4000 m, while along the *z* coordinates the approximate range is 100 to 230 m. Hence, we can assume that during the flying period the pigeon moves in *xy*‐plane—comparatively little motion in the *z*‐direction. Notice that in Figure 2f, the sharp changes along the *z* coordinates occur when the pigeon starts flying or is landing.

**Table 2:** Homing flight: Ranges of *x*,* y* and *z* components of position, velocity and acceleration

Position m	*x*	*y*	*z*
Range	2,900→9,100	2,000→14,000	100→230
Velocity m/s	$\dot{x}$	$\dot{y}$	$\dot{z}$
Range	−18→15	−18→15	−2→2
Acceleration m/s${}^{2}$	$\ddot{x}$	$\ddot{y}$	$\ddot{z}$
Range	−8→8	−8→8	−2→2

Figure 2c represents the velocity estimated by Nagy et al. (2010), it confirms our conclusion as it clearly demarcates the flying and nonflying periods. Moreover, our ad hoc criterion for flight is consistent with these data: A pigeon is deemed to be flying if its velocity magnitude is greater than 4m/s. Furthermore, a comparison among the ranges of the velocity components (Table 2) validates our assumption of flying in the *xy*‐plane. However, in the last quarter of the estimated velocity data, there appear sudden spikes, when no flying motion is supposed to occur (compare Figure 2b,d,f with 2c). This raises some uncertainty over the validity of the estimation method used in (Nagy et al., 2010).

Additionally, Figure 2e,g shows the dynamic acceleration and its *x* component as estimated based on the GPS position data. Again the sharp spikes contribute to our unease over these estimations, especially when some calculated accelerations appear to exceed 80 ${\text{m/s}}^{2}$ (or 9 g). Consequently, it is important to determine to what extent we can rely on these data for further investigation, particularly the GPS‐recorded position data and the estimated acceleration data. Therefore, in the following section, we introduce the main subject of this article: a tracking methodology using the shadowing filter. This method will enable us to estimate the corresponding acceleration of the tracked positions; then, a comparison will be given to check the reliability of the data and our estimates.

### 2.2 Object tracking methodology

In this section, we introduce a method of tracking a moving object, that is, finding the closest plausible and realistic trajectory to noisy observations of the target's position. This is exactly our situation as the GPS devices only recorded data of the pigeons’ positions. The reason why we choose this tracking approach, rather than standard filtering (Jazwinski, 1970), is due to the inaccuracies in the data as discussed in the previous section. We see two major problems with the data at hand: (1) a large number of data points are not recorded but interpolated, due to the GPS device's failure, and (2) some of the estimated velocities and accelerations have unreasonably large values. In particular, acceleration is of great importance because it will be used later to verify our estimated forces that keep the flock together. These two problems, coupled with standard particle or Kalman filter approaches, will lead to systematic biases in the filtered signal. The assumptions driving the shadowing filter approach will avoid these problems.

The tracking methodology we use is based on the idea of the shadowing filter (Stemler & Judd, 2009), which solves the problem from a dynamical system point of view. However, the data were collected and previously analyzed using Hierarchical models (Nagy et al., 2010), which approach the problem from a statistical point of view (Xu, Kattas & Small, 2012). As any object moves under Newton's laws, our methodology aims to find the closest Newtonian trajectory to the observed positions. The robustness of our approach is that it requires only a minimum data length, which means we can exclude the missing GPS data points and still implement our tracking methodology successfully. Moreover, our method enables us to estimate the acceleration based on Newton's laws which we can trust and are used in our latter study.

This tracking method has been introduced previously in (Zaitouny, 2012; Judd, 2015). However, here we provide a brief summary of our methodology including source code (Zaitouny, 2016) and suggestions for appropriate usage. Our objective is to track the position of a point object moving in one dimension, given a sequence of noisy observations. Let $y_{i}\in R$ be the real states, let $P_{i}\in R$ be the noisy observation of its position at time $t_{i}$ for *i* = 0,…,*n* and $\sigma_{i}^{2}\in R$ be the variance of the observational error. The object's dynamics is modeled by its observed position $P_{i}\in R$ , velocity $\nu_{i}\in R$, and constant acceleration $a_{i}\in R$ for $t_{i}\leq t\leq t_{i+1}$. Our goal is to estimate $p_{i}\in R$ close to $y_{i}$. We will minimize the total square error ($\sum_{i=0}^{n}\sigma_{i}^{-2}{\left\|P_{i}-p_{i}\right\|}^{2}$). We assume that the acceleration is constant for one time interval ($T_{i}\;=\;t_{i+1}-t_{i}$) and is bounded over the entire trajectory by the relation ($\sum_{i=0}^{n-1}T_{i}a_{i}^{2}\;\leq \;\left(t_{n}-t_{0}\right)\xi^{2}$). In addition, we assume Newton's laws and Galilean coordinate transformation; therefore, we have two additional constraints:

$$p_{i+1}-p_{i}=\frac{1}{2}a_{i}T_{i}^{2}\;+\;\nu_{i}T_{i},$$

$$\nu_{i+1}-\nu_{i}\;=\;a_{i}T_{i}.$$

We can solve this problem using the Lagrange multipliers method (Press, 2007). An appropriate Lagrange function for our tracking problem is as follows:

(1) $$L\;=\;\frac{1}{2}\sum_{i=0}^{n}\sigma^{-2}{\left\|P_{i}-p_{i}\right\|}^{2}$$

(2) $$+\sum_{i=0}^{n-1}\lambda_{i+1}\left(p_{i+1}-p_{i}-\frac{1}{2}a_{i}T_{i}^{2}-\nu_{i}T_{i}\right)$$

(3) $$+\sum_{i=0}^{n-1}\mu_{i+1}\left(\nu_{i+1}-\nu_{i}-a_{i}T_{i}\right)$$

(4) $$+\;\eta \left(\sum_{i=0}^{n-1}T_{i}a_{i}^{2}-\left(t_{n}-t_{0}\right)\xi^{2}\right),$$

where expression (1) is the total square error we want to minimize, expression (2) is related to the constraints from Newton's first law, the term (3) expresses the constraints from Newton's second law, and (4) represents the acceleration constraint. For (*i* = 1,…,*n*), $\lambda_{i}\in R$, $\mu_{i}\in R$, and η ∈ **R** are our Lagrange multipliers.

The solution occurs where the partial derivatives are zero:

(5) $$\frac{\partial L}{\partial p_{i}}\;=\;\frac{\partial L}{\partial \nu_{i}}=\frac{\partial L}{\partial a_{i}}=\frac{\partial L}{\partial \lambda_{i}}=\frac{\partial L}{\partial \mu_{i}}=\frac{\partial L}{\partial \eta }=0$$

We solved the system using matrix forms and singular value decomposition (Golub & Loan, 1984) method to obtain the least‐squares approximate solution *p* for the observed positions *P* and their corresponding acceleration for a given smoothing parameter η. For more details, see the Supplementary Materials. Our proposed method is computationally fast, efficient and allows for effective optimization. The provided code (Zaitouny, 2016) demonstrates the applicability of our method to noisy data with missing observations and irregularly sampled trajectories. Although the code is provided for scalar case, it is easy to extend to multiple dimensions or multiple objects. Alternatively, our method is sufficiently robust that the error correlation among different dimensions can be ignored (Zaitouny, 2012). That is, one can treat a high‐dimensional problem as independent scalar problems.

## 3 Results

In this section, we will implement our methodology for a single flying pigeon (both in free flight and in homing) to verify our model performance and judge the reliability of the data set. The tracking will be applied to the flight period (the most interesting and nontrivial behavior pattern). We down‐sample with a variety of different decimation factors to illustrate the robustness of our algorithm when presented with more sparsely sampled time series. We will show how powerful our tracking technique is, and how it works successfully even when we exclude the data points that the GPS device failed to observe.

In order to investigate the model performance and optimize its parameters, we compute the root‐mean‐square error (RMS) along the entire trajectory to be our measurement:

(6) $$E=\sqrt{\frac{1}{S}\sum_{i=0}^{S}\left(\right\|X_{i}-x_{i}{\|}^{2}\;+\;\|Y_{i}-y_{i}{\|}^{2}\;+\;\|Z_{i}-z_{i}{\|}^{2})}$$

where *S* is the size of the down‐sample observations, $X_{i},Y_{i}$ and $Z_{i}$ are the observed coordinates, and $x_{i},y_{i}$, and $z_{i}$ are our estimated coordinates.

### 3.1 Tracking free‐flying pigeon

In this subsection, we will consider one pigeon from the free‐flying flock where the free‐flying pattern has a length of 1,500 points. Specifically, we will consider a subtrajectory of pigeon *A* shown in the previous section (Figure 1) and focus on the period from [8,500, 10,000) which is obviously a period of flying. While in flight our tracking methodology will be implemented in three different situations: (1) taking the whole subtrajectory (1,500 points), that is, the time resolution is 0.2 s; (2) taking each fifth point, which means a sub‐sub‐trajectory of length (300 points) and time resolution 1 s; and (3) we extend the considered subtrajectory to include 200 extra points, then down‐sample it to a time resolution of 2 s (170 points). The reason behind choosing different down‐sample sizes is to show the robustness of our tracking method and illustrate how the missing data points will not affect the performance of our tracking technique.

As we only aim to explore our tracking method performance, we can use a simple numerical scheme. We take a broad parameter sweep with η between 10 and $10^{-6}$ and calculating *E* (Equation (6) for both position and acceleration) for different time resolutions as described above.

**Figure 3:**
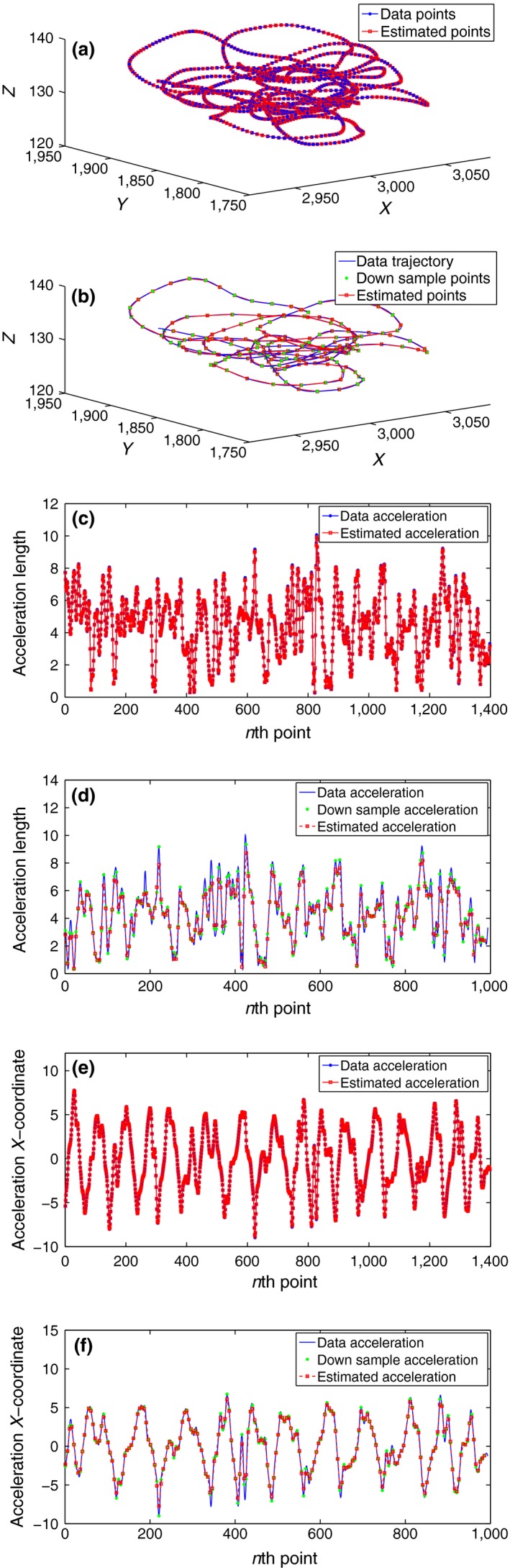
Free Flight: Tracking pigeon A position and acceleration with optimal values of η corresponding to two different time resolution 0.2 s and 1 s. (a) Whole trajectory: $\eta \;=\;5\;\times \;10^{-6}$, Δ T = 0.2 s. (b) Whole trajectory: $\eta \;=\;5\;\times \;10^{-5}$, Δ T = 1 s. (c) Acc. magnitude: $\eta \;=\;5\;\times \;10^{-6}$, Δ T = 0.2 s. (d) Acc. magnitude: $\eta \;=\;5\;\times \;10^{-5}$, Δ T = 1 s. (e) Acc. x‐component: $\eta \;=\;5\;\times \;10^{-6}$, Δ T = 0.2 s. (f) Acc. x‐component: $\eta \;=\;5\;\times \;10^{-5}$, Δ T = 1 s

Table 3 shows the results of our numerical experiments. Where “*E*‐position” is calculated from the position data points and our estimated positions, and “*E*‐acceleration” is the root‐mean‐square error between the acceleration from the data and the acceleration we estimate via our model. It is apparent from the table that there are optimal values of the smoothing parameter η for each time resolution. With time resolution as 0.2 s, it has been found that the optimum value of η lies in the interval ($10^{-6},10^{-5}$) with *E*‐position $\approx 7.3510\;\times \;10^{-5}\;m$ and *E*‐acceleration $\approx \;0.0469\;{\text{m/s}}^{2}$. The corresponding results are illustrated in Figure 3a,c,e, where the smoothing parameter η is chosen to be $5\;\times \;10^{-6}$. Figure 3a shows a comparison between our position tracking estimations and the data, it represented for the whole trajectory. It can be easily noticed how close the estimations are to the observations. Figure 3c,e compare between the data's acceleration and our tracking estimated acceleration, the former shows for the acceleration's magnitude, while the latter shows the acceleration *x* component. It is clear that our acceleration approximations are very close to those of the original data—except at some sharp spikes where our estimations have eliminated these spikes, which is more realistic.

**Table 3:** Numerical experiments results of free flight to determine the optimum value of η for different time resolution. Given are the root‐mean‐square errors for position (E‐Pos. in m) and acceleration (E‐Acc. in per m/s${}^{2}$)

η	Time resolution = 0.2 s		Time resolution = 1 s		Time resolution = 2 s	
	E‐Pos.	E‐Acc.	E‐Pos.	E‐Acc.	E‐Pos.	E‐Acc.
10	5.57	2.11	13.9	3.07	19.1	3.37
5	3.43	1.82	9.58	2.66	14.0	2.95
1	1.09	1.33	3.37	1.94	5.35	2.15
0.5	0.68	1.14	2.09	1.72	3.33	1.92
0.1	0.23	0.74	0.73	1.30	1.17	1.56
0.05	0.14	0.59	0.47	1.14	0.80	1.45
0.01	0.042	0.31	0.18	0.85	0.38	1.23
$5\;\times \;10^{-3}$	**0.025**	**0.23**	**0.12**	**0.75**	**0.28**	**1.16**
$10^{-3}$	0.0062	0.12	0.049	0.60	0.14	1.05
**5** **×** $10^{-4}$	0.003	0.09	0.036	0.56	**0.12**	**1.03**
$10^{-4}$	7.8$\;\times \;10^{-4}$	0.058	0.021	0.498	2.34	2.41
**5** **×** $10^{-5}$	4.2 $\times \;10^{-4}$	0.052	**0.017**	**0.48**	16.9	16.4
$10^{-5}$	1.1 $\times \;10^{-4}$	0.047	2.78	10.6	235	232
**5** **×** $10^{-6}$	**7.35** **×** $10^{-5}$	**0.047**	17.0	65.5	407	403
$10^{-6}$	1.0 $\times \;10^{-4}$	0.047	222	880	707	702
5 $\times \;10^{-7}$	2.1 $\times \;10^{-4}$	0.05	432	1.7 $\times \;10^{3}$	766	760
$10^{-7}$	0.051	48.4	990	3.9 $\times \;10^{3}$	818	811

Bold values highlight the optimal values of the smoothing parameter and the corresponding minimal errors.

While for time resolution 1 s, it has been found the minimum of *E*‐position ≈ 0.0171 m and the minimum of *E*‐acceleration $\approx \;0.4838\;{\text{m/s}}^{2}$ occur at an optimal value of η inside the interval ($10^{-5}$,$10^{-4}$). The increase in the minimum values of *E*‐position and *E*‐ acceleration comparing with their values in 0.2 s time resolution is not surprising, because the number of points used for the tracking with 1 s time resolution is much smaller. Figure 3b,d,f shows the results of our tracking filter with this time resolution and a smoothing parameter $\eta \;=\;5\;\times \;10^{-5}$ lies in the optimal interval. In these figures, the solid blue lines refer to the whole data trajectory from which we extract the down‐sampled version, the down‐sample points which are used in the filter are indicated as green stars, the outcomes of our tracking using the down sample are represented as red squares. Figure 3b compares the positions, we observe that our filter still gives very good results even with using just a down sample and not the whole data set. While Figure 3d,f compares the acceleration estimated using the down sample with data's acceleration, here the mismatching at the spikes is much more noticeable.

Finally, for time resolution of 2 s, as expected we found larger minimum *E*‐position ≈ 0.1163 m and *E*‐acceleration $\approx \;1.0261\;{\text{m/s}}^{2}$ corresponding to an optimal value of η in the interval ($10^{-4}$, $10^{-3}$). However, our position estimations are still very close to the observations, as well as the estimated accelerations despite the expected gaps occurring at the sharp spikes.

### 3.2 Tracking homing flying pigeon

Following from the previous subsection, we now apply our shadowing filter to homing flight. That is, we will choose pigeon *A* from homing flight and consider a subtrajectory of length 1,500 points of its flying pattern; particularly, we consider the period of points [2,500, 4,000] of its trajectory. When analyzing the remaining flying segments, we obtained similar results. Along this subtrajectory, we implement our tracking filter for the three different situations (time resolution = 0.2 s, 1 s and 2 s).^2^ The implementation has been performed for the same broad parameter sweep as above.

Table 4 illustrates the outcomes of these numerical investigations. It has been found for 0.2 s time resolution that the optimum value of the smoothing parameter η lies in the interval (5 $\times \;10^{-6}$, 5 $\times \;10^{-5}$), where the approximation of the corresponding minimum errors are *E*‐position$\approx 9.4705\;\times \;10^{-5}\;m$ and *E*‐acceleration $\approx \;0.0389\;{\text{m/s}}^{2}$. For time resolution = 1 s, it can be seen from Table 4 that the minimum of *E*‐position ≈ 0.0122 m and the minimum of *E*‐acceleration $\approx \;0.3733\;{\text{m/s}}^{2}$ correspond to an optimal value of the smoothing parameter η lies in the interval ($5\;\times \;10^{-5}$, $5\;\times \;10^{-4}$). Lastly, as expected for 2 s time resolution, the minimum values of the errors are slightly increased such that *E*‐ position ≈ 0.0564 and *E*‐acceleration ≈ 0.6091, which occur at an optimal value of η inside the interval ($10^{-4}$, $10^{-3}$). Note that, by comparing these results with the results from free flying in the previous section, we find that the tracking filter's performance is consistent between these two very different behaviors.

**Table 4:** Numerical experiments results of homing flight to determine the optimum value of η for different time resolution. Again the errors of the position (E‐Pos. m) and the acceleration (E‐Acc. ${\text{m/s}}^{2}$) are given

η	Time resolution = 0.2 s		Time resolution = 1 s		Time resolution = 2 s	
	E‐Pos.	E‐Acc.	E‐Pos.	E‐Acc.	E‐Pos.	E‐Acc.
10	3.09	1.73	6.02	2.19	6.03	1.60
5	2.13	1.54	4.65	2.03	4.41	1.52
1	0.85	1.10	2.22	1.61	2.15	1.34
0.5	0.56	0.92	1.53	1.42	1.61	1.25
0.1	0.19	0.56	0.59	1.02	0.78	1.02
0.05	0.11	0.44	0.39	0.88	0.56	0.94
0.01	0.033	0.24	0.13	0.62	0.27	0.77
$5\;\times \;10^{-3}$	**0.019**	**0.18**	**0.085**	**0.55**	**0.19**	**0.71**
$10^{-3}$	0.005	0.094	0.033	0.440	0.08	0.63
$5\;\times \;10^{-4}$	0.003	0.072	0.024	0.41	**0.056**	**0.61**
$10^{-4}$	5.8 $\times \;10^{-4}$	0.05	**0.012**	**0.373**	11.0	10.5
$5\;\times \;10^{-5}$	3.0 $\times \;10^{-4}$	0.042	0.014	0.37	80	78
$10^{-5}$	**9.5** **×** $10^{-5}$	**0.039**	13.2	50.4	1.1 $\times \;10^{3}$	1.1 $\times \;10^{3}$
$5\;\times \;10^{-6}$	1.2 $\times \;10^{-4}$	0.040	81	312	1.9 $\times \;10^{3}$	1.9 $\times \;10^{3}$
$10^{-6}$	4.1 $\times \;10^{-4}$	0.056	1.1 $\times \;10^{3}$	4.2 $\times \;10^{3}$	3.4 $\times \;10^{3}$	3.3 $\times \;10^{3}$
$5\;\times \;10^{-7}$	$10^{-3}$	0.11	2.1 $\times \;10^{3}$	8.2 $\times \;10^{3}$	3.6 $\times \;10^{3}$	3.6 $\times \;10^{3}$
$10^{-7}$	2.45	230	4.7 $\times \;10^{3}$	1.$\;\times \;10^{4}$	3.9 $\times \;10^{3}$	3.9 $\times \;10^{3}$

**Figure 4:**
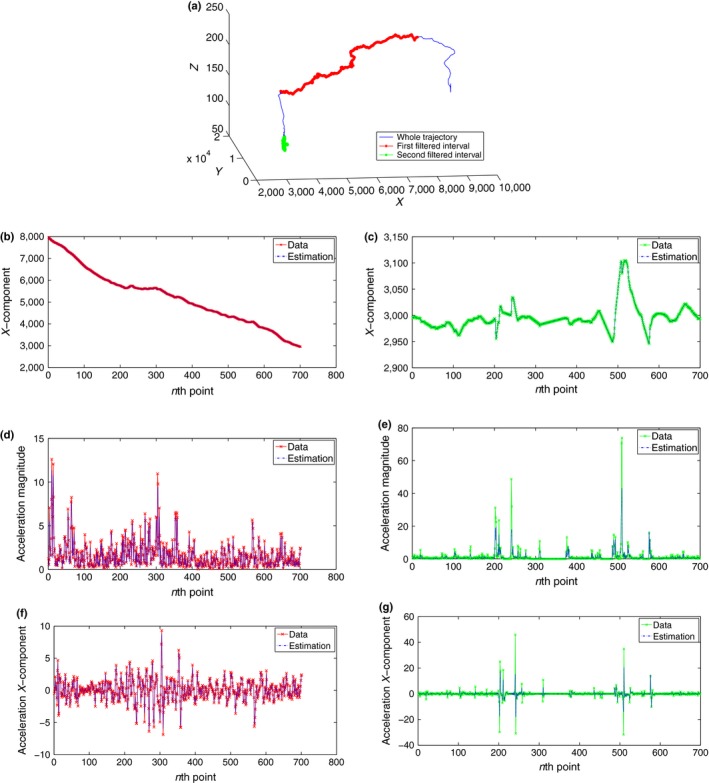
Homing Flight: Tracking pigeon A position and acceleration with $\eta \;=\;10^{-4}$ and time resolution = 1 s. (a) Position: Whole trajectory. (b) Position: *X* coordinates. (c) Position: *X* coordinates. (d) Acceleration: magnitude. (e) Acceleration: magnitude. (f) Acceleration: *X* component. (g) Acceleration: *X* component

Additionally, to show our results graphically, Figure 4 represents comparisons between our filter estimations and data observations for both positions and accelerations along two different periods—the filter has been applied for $\eta \;=\;10^{-4}$ and time resolution = 1 s. In order to confirm that our tracking filter estimations are closer to reality than the data observations, especially for acceleration, the two periods have been chosen as follows (see Figure 4a): The red down sample includes 700 points of a flying interval (each fifth point of the interval [1,500, 5,000]). While the green down sample has been chosen purposely to include an episode of rather doubtful acceleration in the data set as mentioned before in subsection (3.2), this down sample includes 700 points of the last quarter of the trajectory (each fifth point of the interval [10,000, 13,500]). It can be seen from Figure 4b,d,f how the estimations are close to the observations for both position and acceleration along the flying period. On the other hand, for the doubtful region, we can notice from Figure 4c that the matching between our position tracking estimations and data observations is almost perfect. However, Figure 4e,g shows how our estimated accelerations avoid the unrealistic states shown in the last quarter of the data set, where the data set includes accelerations around 80 ${\text{m/s}}^{2}$, while our estimations of acceleration does not exceed 40 ${\text{m/s}}^{2}$ , which supports our assumption that our tracking estimated accelerations are closer to reality than the data's accelerations.

### 3.3 Reliability of the data

The data set we used is a filtered GPS data with 0.4 $m^{2}$ error variance due to the device inaccuracy; moreover, as mentioned before, the velocity and acceleration provided in the data are calculated using the GPS position information. Therefore, it is important for our further investigations to verify the reliability of these data especially the acceleration and optimize the performance of our filter and the significance of its impact on the calculation of acceleration. To do that, we propose two numerical experiments. The first one is to optimize the smoothing parameter η according to the error variance of the GPS device using simulation data. Such an optimization is needed because we do not know the true trajectory of the bird but only the recorded position data. The second numerical experiment determines the minimum trajectory length required to implement our tracking filter. In addition, we give some comparison between our method and a sliding window filter.

#### 3.3.1 Simulation data optimization

In our previous numerical investigations, we found that there exists an optimal value of the smoothing parameter η that implies the closest estimations to the data observations (c.f. Tables 3 and 4). However, we now ask whether this achieves our objective, or can we do something better? In fact, as we only have noisy observations from the GPS device, we should aim to find the closest (most plausible) trajectory to the unknown reality. Plausibility in our case means that the trajectory we are looking for is consistent with our model of the flying pigeon. Therefore, we will use a scaling relationship between the error variance and the corresponding optimal value of the smoothing parameter η which (we claim) gives the closest trajectory to reality from the simulation data. The idea can be described briefly as follows: we generate a *true trajectory* and for each noise level in a certain range we also generate 100 noisy trajectories that we use as observations. Obviously the noise level range is chosen such that it fits with the measurement uncertainty of the equipment used in the real experiment. Starting from a broad parameter sweep of η, we estimate a trajectory for the observations. Measuring the error between the 100 estimated sequences and the true trajectory allows us to narrow our parameter sweep around the minimum error. Several sweeps and consequent limiting of the range of η around the new minimum results in a simple optimization of the η parameter. It has been found in (Zaitouny, 2012; Zaitouny, Stemler & Judd, In press) that the best value of η is proportional to the noise's standard deviation cubed. An approximated relationship for high sampling rates is found to be

(7) $$\eta_{b}\;\approx \;0.046{\sqrt{\beta }}^{3}\;+\;0.054,$$

where $\eta_{b}$ is the desired optimal value of η and β is the noise's variance.

**Figure 5:**
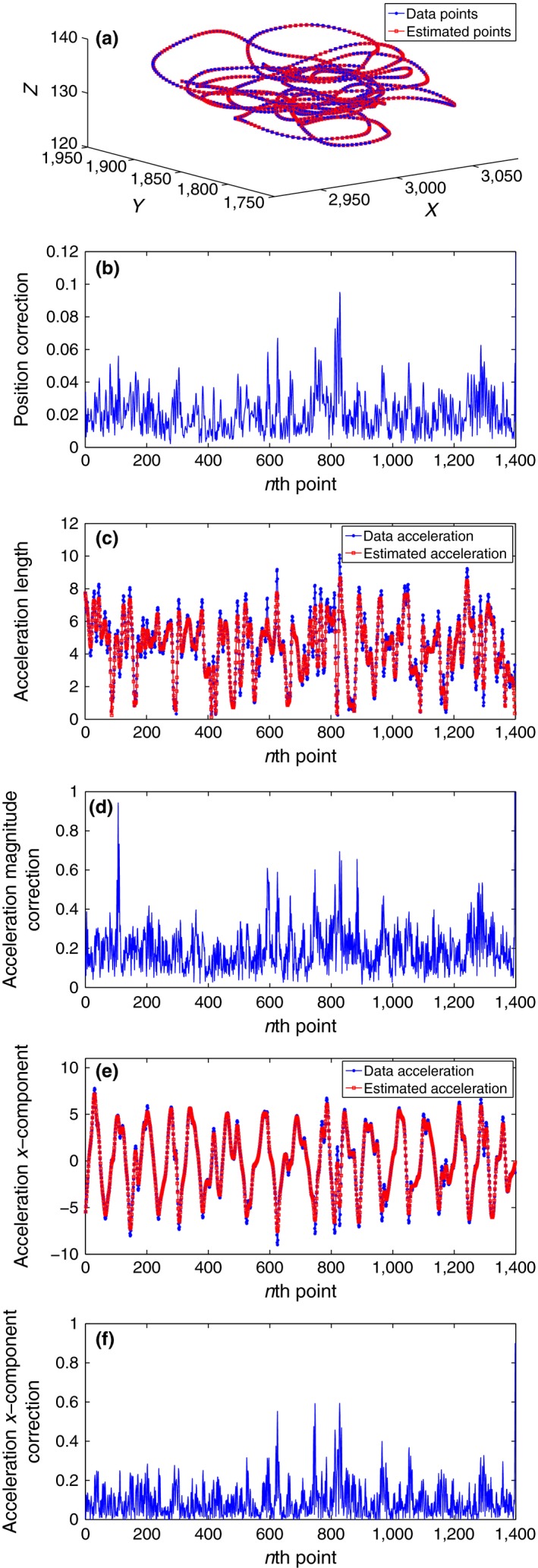
Free flight: Tracking pigeon A position with an approximation of the best value of $\eta \;=\;5\;\times \;10^{-3}$ gives the closest estimates to reality and time resolution = 0.2 s. (a) Whole trajectory. (b) Trajectory corrections. (c) Acceleration magnitude. (d) Acceleration corrections. (e) Acceleration *X* coordinates. (f) *X* acceleration corrections

Such a relationship allows us to no longer optimize η based on some interpretation of being close to the observations (as we have done so far for example in Tables 3 and 4), but instead choose η according to the device's error variance of 0.4 $m^{2}$. Accordingly, we can conclude that implementing our tracking technique on this data set with a value of the smoothing parameter η ≈ 0.05 will result in estimations closer to the reality. In Table 3, we can find *E*‐position = 0.14 m and *E*‐acceleration = 0.59 m/s^2^ corresponding to η = 0.05, which are further from the observations but according to Equation (7) should be close to the true trajectory. Figure 5 represents the results of this application for the free flying pigeon *A*'s subtrajectory of length 1,500 points and time resolution = 0.2 s. Figure 5a shows how the position estimations are close to observations, the differences are not obvious, but Figure 5b shows that corrections varying between 0 and 0.1 m have been observed. Actually an improvement occurred at the spikes in the data. This improvement can be seen better when we compare the estimated acceleration (Figure 5c,d). It is noticeable how our tracking filter, corresponding to the optimum η = 0.05, tries to avoid strong and sharp changes in acceleration and therefore gives more realistic estimates. The corrections in the acceleration magnitude vary between 0 and 1 ${\text{m/s}}^{2}$. Figure 5e,f show the estimations and the corrections of the acceleration's *x* component in order to emphasis on the significant improvements made by our tracking technique. We see how our filter treats the sudden and strong changes in acceleration, and how it modifies the estimations to be closer to real dynamics.

#### 3.3.2 Windowing test

The widowing test is a useful procedure to address questions of predictability and data requirements arising in real‐world applications. The test was introduced in (Stemler & Judd, 2009), and the basic idea of this test is to apply the shadowing filter to data sequences of increasing length. By examining the filter's convergence, we can find a minimum measurement or window length that is needed to get appropriate approximations. Consistency of convergence beyond that minimum length also provides a good guide to reliability of the filter in the particular application setting.

A basic application of the widowing test is to determine a minimum window length of observations that is required to obtain a good tracking of the positions of the travelled object, that is, one is interested in a good convergence along the whole trajectory. The widowing test can be applied to real measurements or artificial data. In our case, we do have a real data set but we do not know the true states of the pigeons; therefore, we will use the windowing test to verify the reliability of the data set.

For the first application when the true states are unknown, the widowing test is applied as follows. Given a long trajectory of noisy observations $P_{N}\;=\;\left(P_{1},\cdots ,P_{N}\right)$, apply the shadowing filter at the optimal parameter η to the length *n* subtrajectories $P_{n}\;=\;\left(P_{N-n+1},\cdots ,P_{N}\right)$, for 0 ≤ *n* ≤ *N*, to obtain approximated trajectories $Q_{\eta ,n}\;=\;\left(q_{N-n+1},\cdots ,q_{N}\right)$. As *n* is decreased the lengths of the subtrajectories are decreased, therefore, we compare the estimated subtrajectories $Q_{\eta ,n}$ to the corresponding observations of $P_{N}\;=\;\left(P_{1},\cdots ,P_{N}\right)$. That is, we compare the distances $D_{j,n}\;=\;\|P_{N-j}-q_{N-j,\eta ,n}\|$ for 0 ≤ *j* < *n*.

**Figure 6:**
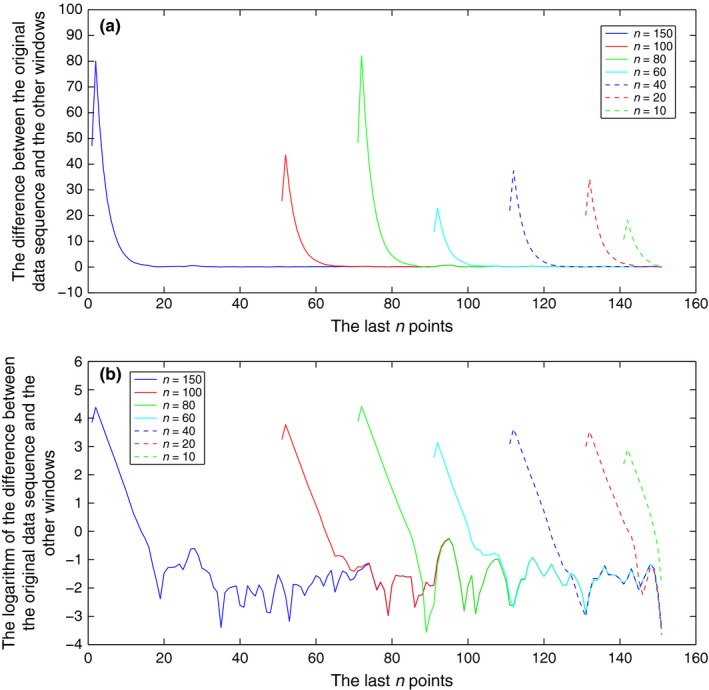
Application of the windowing test: Estimates for different length of observation sequences. (a) $D_{j,n}$ as a function of the position. (b) The logarithm of $D_{j,n}$ as a function of the position

Figure 6 represents an application of the widowing test using an initial down‐sample sequence from the recorded flight pattern with time resolution 2 s and a length of 200 observations. We compare the estimation of this 200 observation long sequence with the ones resulting from subtrajectories of lengths *n* = 150, 100, 80, 60, 40, 20, 10. For all cases, we used the optimal parameter η = 0.05. Each line in Figure 6a plots the distances $D_{j,n}$ between observations $P_{N}$ and corresponding states $Q_{\eta ,n}$. Observe the convergence of the $P_{N}$ to $Q_{\eta ,n}$, the convergence at the last state^3^ occurs for all *n* ≥ 20; therefore, we conclude that the minimum length of the observational trajectory that is required for position tracking is *n* = 20 and therefore about 40 s.

Figure 6b represents the logarithm of the previous distances. From this figure, we can observe that the minimum error in the observed data set is about 0.3 m.

Moreover, according to Figure 1 by observing the nonflying positions, we can say that the data have a maximum error of 3 m. Therefore, we can confirm that the position data set we have is sufficiently reliable (error ∈  (0.3, 3) m) and it can be used to extend our work. However, we observe above that the acceleration provided with the data set is not reliable. Therefore, it is more appropriate to use our tracking filter to estimate new accelerations closer to reality to be used in our further investigations.

## 4 Discussion

We have shown in (Zaitouny, 2012; Zaitouny et al., submitted, under review) that our tracking methodology (1) is able to minimize the error and can be optimized corresponding to the observational error and time resolution, (2) is easy to adjust for one dimension or higher dimensions, (3) is robust enough to consider or ignore the error correlation, (4) works successfully for regular or irregular time resolution, (5) is capable to be extended to track rigid bodies and (6) is able to reconstruct the full dynamical state space only from position observations. In this article, we have seen our shadowing filter tracking approach is able to track the dynamics of an individual pigeon very well and applying the filter improved the quality of the data. Conversely, we should also justify the introduction of this new filter when popular alternatives are already well accepted. In the literature, one can find a variety of filter methods, including variational filters (Swanson, Vautard & Pires, 1998) Kalman filters (Bar‐Shalom, Li & Kirubarajan, 2001; Hartikainen, Solin & Särkkä, 2011), and other statistical filters (Jazwinski, 1970). The variational filters start to be no longer useable if the time series get too long (Stemler & Judd, 2009), while the other two approaches are rather widely used nowadays. These statistical filters are both sequential, that is, instead of using all the available time series information at once the next state is optimized based on the value of the current state. As we have seen the shadowing filter uses the time series as a whole and optimization is done not on neighboring states but on the complete time series. We argue that this leads to better results as it more appropriately used all the available information. Note that the shadowing filter framework has already been tested against a variety of alternative filter techniques, including all the usual suspects (see Judd, 2003; Judd & Stemler, 2009; Stemler & Judd, 2009), and shown to outperform. While it seems to be a major drawback that we need a minimum time series length to apply our filter, it should be noted that this minimum length is similar to the length required for good estimates from sequential filters like the Kalman filter or other Bayesian filters. In addition, we want to also point out that while other filters lead to good position approximations and therefore can provide good tracking results, the other filters require some postprocessing to reconstruct the full phase‐space consisting of position, velocity and acceleration. On the other hand, for the specific tracking application of these filters, in (Zaitouny et al., submitted, under review), a direct comparison has been conducted among our method, Kalman , extended Kalman, and particle filters’ tracking approaches (Gustafsson et al., 2002; Gustafsson, 2010; Hartikainen et al., 2011). The results support the superiority of our method in terms of performance (accuracy) and computational complexity (speed). In the following subsection, we introduce an additional simple comparison with the sliding average filter.

### 4.1 Comparison with a sliding average filter

The simplest possible sequential filter is the sliding average filter (Wei, 1994). In this filter, the position is stimated by averaging *n*‐observations to get an estimate of the current state, that is $p_{i}\;=\;1/n\sum_{j=i-(n-1)/2}^{i+(n-1)/2}P_{j}$. If the noise level is small and unbiased, this filter gives reasonable approximations of the true position. For our experiment, we used the data from the homing flight. Given that these data have a high temporal resolution (0.02 s.) and that the measurement noise is comparably small, we can directly apply this filter and we do so with *n* = 5. As with the Kalman filters or any other sequential filter reconstruction of the full phase‐space requires us to numerically differentiate the position data, so that $v_{i}\;=\;\left(p_{i+1}-p_{i}\right)/\Delta t$ and $a_{i}\;=\;\left(v_{i+1}-v_{i}\right)/\Delta t$. It is well known that even if there is very little noise in the position estimates $p_{i}$, the derivatives will amplify the noise. In addition sliding average filters tend to underestimate the turning points of the time series.

**Figure 7:**
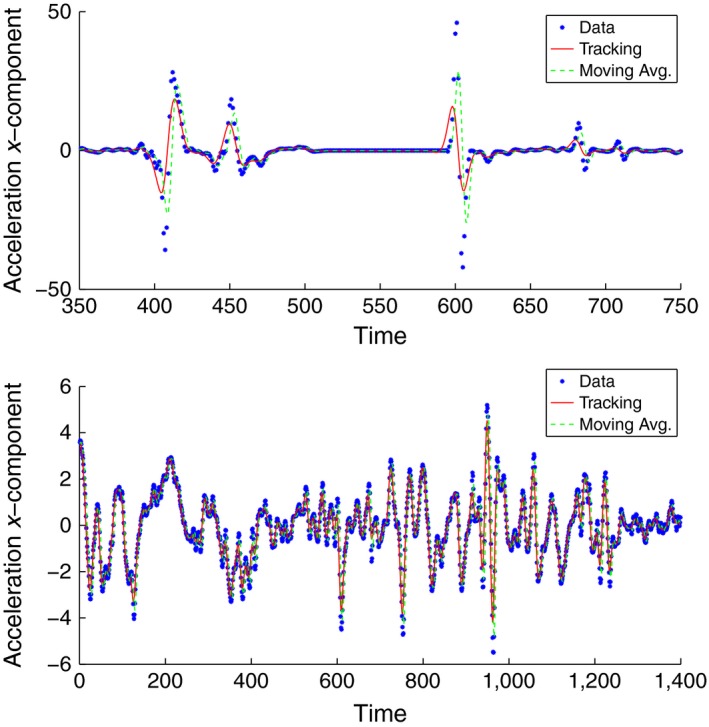
Comparison between the shadowing filter and the sliding average filter for two segments of the homing flight. In the upper panel, we see that the sliding average filter leads to unreasonable high spikes in the acceleration, while in the lower panel, we find that the turning points of the time series are underestimated by this filter

In Figure 7, we show the comparison between the two methods. As expected, the sliding average filter performs much worse than the shadowing filter (η = 0.05). Not only does this lead this method to several unreasonable high values for the acceleration (for example, seven spikes in the upper panel of Figure 7) but in addition the turning points in the lower panel of Figure 7 are underestimated. As mentioned above, this result does not only apply to this particular filter but is a generic property of all sequential filters, that require numerical differentiation to reconstruct the full phase‐space. On the other hand, the shadowing filter is not prove to this problem, because the full phase‐space is reconstructed based on the whole time series and a postprocessing therefore is not needed.

## 5 Conclusion

In this article, we have shown how the shadowing filter framework can be applied to track the motion of a pigeon. Due to the measurement error associated with the GPS tracking device, significant data analysis is needed to get a better understanding of the motion of such targets (birds pose a particularly challenging test case for tracking algorithms). Moreover, we have shown that our method does not need such a high sampling rate and can deal with down‐sampled data having a 10 times longer resolution easily. In addition—as the code shows (Zaitouny, 2016)—the time resolution does not have to be regular and therefore our algorithm can deal with irregularly sampled trajectories. In fact, in several applications mostly with coarse time resolution and also missing data our method provides an excellent alternative to the traditional statistical filters. In addition, we have shown using the windowing test that our method is also applicable for much shorter time series than the one at hand.

It is important that our tracking method does not require any input in terms of biological parameters. Just making the (valid) assumption that pigeons obey Newtonian mechanics is sufficient. This allows us to improve the data quality without any questionable assumptions that might underlay a biological model.

While we see our contribution as a first, important step to improve the data quality before modeling the flock's dynamics, it is clear that our method could be applied in other problems too. While implementation of our filter for already existing GPS data (Steiner et al., 2000; Gremillet et al., 2004; DeCesare et al., 2005) is an obvious application, it should be noted that our method could also be applied to position data resulting from visual methods of tracking animals (Ballerini, Cabibbo, Candelier, Cavagna, Cisbani, et al., 2008; Gautrais, Ginelli, Fournier, Blanco, Soria, et al., 2012; Bhagavatula, Claudianos, Ibbotson & Srinivasan, 2014; Boos, Pritz, Lange & Belz, 2014).

## Conflict of Interest

None declared.

## Data Accessibility

We gratefully acknowledge the data provided by M. Nagy, Zs. Akos, D. Biro and T. Vicsek, the authors of (Nagy et al., 2010).

## Notes

1 Direct differentiation estimates the velocity as the difference (distance) between consecutive positions divided by the corresponding time interval; similarly, the acceleration is the difference between consecutive velocities divided by the time interval. The primary problem with direct differentiation is that numerical differencing will amplify the effect of noise.

2 Again with time resolution = 2 s, we extend the subtrajectory to 1,700 points

3 Remember that our filter show large errors at the very beginning of the sequence.
