# Supplementary material for: Tracking a single pigeon using a shadowing filter algorithm
Source: Ecol Evol. 2017 May 11;7(12):4419–31. doi: 10.1002/ece3.2976 (PMC5478088; doi:10.1002/ece3.2976)
Supplement: Supplementary file 1 [file ECE3-7-4419-s001.pdf]

Supplementary Materials

Details on the tracking algorithm

For our problem (2.1-2.4) the partial derivatives are:

$$\frac{\partial L}{\partial p_i} = 0 = \begin{cases} -\sigma_0^{-2}(P_0 - p_0) - \lambda_1, & i = 0 \\ -\sigma_i^{-2}(P_i - p_i) + \lambda_i - \lambda_{i+1}, & 0 < i < n \\ -\sigma_n^{-2}(P_n - p_n) + \lambda_n, & i = n \end{cases} \tag{1}$$

$$\frac{\partial L}{\partial \nu_i} = 0 = \begin{cases} -\lambda_1 T_0 - \mu_1, & i = 0 \\ -\lambda_{i+1} T_i + \mu_i - \mu_{i+1}, & 0 < i < n \\ \mu_n, & i = n \end{cases} \tag{2}$$

$$\frac{\partial L}{\partial a_i} = 0 = -\frac{1}{2}\lambda_{i+1} T_i^2 - \mu_{i+1} T_i + 2\eta T_i a_i \tag{3}$$

$$\frac{\partial L}{\partial \lambda_i} = 0 = p_{i+1} - p_i - \frac{1}{2} a_i T_i^2 - \nu_i T_i \tag{4}$$

$$\frac{\partial L}{\partial \mu_i} = 0 = \nu_{i+1} - \nu_i - a_i T_i \tag{5}$$

$$\frac{\partial L}{\partial \eta} = 0 = \sum_{i=0}^{n-1} T_i a_i^2 - (t_n - t_0)\xi^2 \tag{6}$$

By combining Eq. (4), we can eliminate ν_i. To do this we multiply Eq. (4) by T_{i-1}, and use the Galilean transformation to have another copy of Eq. (4) with i replaced with $i-1$ and multiply it by T_i as follow:

$$p_{i+1} T_{i-1} - p_i T_{i-1} - \frac{1}{2} a_i T_i^2 T_{i-1} - \nu_i T_i T_{i-1} = 0$$
$$p_i T_i - p_{i-1} T_i - \frac{1}{2} a_{i-1} T_{i-1}^2 T_i - \nu_{i-1} T_{i-1} T_i = 0,$$

Subtracting the two equations results in:

$$p_{i+1}T_{i-1} - p_i(T_i + T_{i-1}) + p_{i-1}T_i - \frac{1}{2}T_{i-1}T_i(a_i T_i - a_{i-1}T_{i-1}) - T_i T_{i-1}(\nu_i - \nu_{i-1}) = 0.$$

Note that from Eq. (5) we have $\nu_i - \nu_{i-1} = a_{i-1}T_{i-1}$, substitution in the previous equation gives:

$$p_{i+1}T_{i-1} - p_i(T_i + T_{i-1}) + p_{i-1}T_i - \frac{1}{2}T_{i-1}T_i(a_i T_i - a_{i-1}T_{i-1}) - T_i T_{i-1}^2 a_{i-1} = 0,$$

and therefore:

$$p_{i+1}T_{i-1} - p_i(T_i + T_{i-1}) + p_{i-1}T_i = \frac{1}{2}T_{i-1}T_i(a_iT_i + a_{i-1}T_{i-1}). \qquad (7)$$

We can combine eqs.(1, 2, 3) to eliminate λ_i and μ_i . To do this we introduce a matrix notation, which simplifies the problem. Moreover, since we consider the linear eqs.(1, 2, 3), we can use the common properties of matrices to solve the linear system. We derive a matrix representation of our problem by defining the following vectors :

$$\begin{aligned}
Y &= (y_0, \cdots, y_n)^T \in \mathbf{R}^{n+1} \\
P &= (P_0, \cdots, P_n)^T \in \mathbf{R}^{n+1} \\
p &= (p_0, \cdots, p_n)^T \in \mathbf{R}^{n+1} \\
\lambda &= (\lambda_1, \cdots, \lambda_n)^T \in \mathbf{R}^{n} \\
\mu &= (\mu_1, \cdots, \mu_n)^T \in \mathbf{R}^{n}; \text{where} \quad \mu_n = 0 \\
a &= (a_0, \cdots, a_{n-1})^T \in \mathbf{R}^{n}
\end{aligned}$$

Additionally, we define T to be the $n \times n$ matrix of zeros with main diagonal $(T_0, \cdots, T_{n-1})$,

$$T_{n\times n} = \begin{pmatrix} T_0 & 0 & \cdots & 0 \\ 0 & T_1 & \cdots & 0 \\ \vdots & \ddots & \ddots & \vdots \\ 0 & 0 & \cdots & T_{n-1} \end{pmatrix},$$

K to be the $(n+1)\times(n+1)$ matrix of zeros with main diagonal $(\sigma_0^{-2}, \cdots, \sigma_n^{-2})$.

$$K_{(n+1)\times(n+1)} = \begin{pmatrix} \sigma_0^{-2} & 0 & \cdots & 0 \\ 0 & \sigma_1^{-2} & \cdots & 0 \\ \vdots & \ddots & \ddots & \vdots \\ 0 & 0 & \cdots & \sigma_n^{-2} \end{pmatrix}.$$

We also define the D matrix as a $(n \times n)$ matrix with zero everywhere except -1 on the main diagonal and 1 on the first lower diagonal,

$$D_{n\times n} = \begin{pmatrix} -1 & 0 & 0 & \cdots & 0 \\ 1 & -1 & 0 & \cdots & 0 \\ 0 & 1 & -1 & \ddots & 0 \\ \vdots & \ddots & \ddots & \ddots & \vdots \\ 0 & 0 & \cdots & 1 & -1 \end{pmatrix}.$$

similarly define E as a $((n+1)\times n)$ matrix that has all entries zero except -1 on the main diagonal and 1 on the first lower diagonal, such that:

$$E_{(n+1)\times n} = \begin{pmatrix} -1 & 0 & 0 & \cdots & 0 \\ 1 & -1 & 0 & \cdots & 0 \\ 0 & 1 & -1 & \ddots & 0 \\ \vdots & \ddots & \ddots & \ddots & \vdots \\ 0 & 0 & \cdots & 1 & -1 \\ 0 & 0 & \cdots & 0 & 1 \end{pmatrix}.$$

Note that these matrices can be expressed as:

$$D_{ij} = E_{ij} = \begin{cases} -1, & i = j, \\ 1, & i = j+1, \\ 0, & \text{otherwise}, \end{cases} \tag{8}$$

where $i = 1, \cdots, n$ for D, while $i = 1, \cdots, n+1$ for E and $j = 1, \cdots, n$ for both matrices.
Using these definitions eqs.(1, 2, 3) can be written respectively as:

$$\begin{aligned} E_{(n+1)\times n}\lambda_{n\times 1} &= K_{(n+1)\times(n+1)}(P_{(n+1)\times 1} - p_{(n+1)\times 1}), & (9) \\ D_{n\times n}\mu_{n\times 1} &= T_{n\times n}\lambda_{n\times 1}; \quad \mu_n = 0, & (10) \\ 2\eta T a &= \frac{1}{2}T^2\lambda + T\mu. \end{aligned}$$

Where the last equation can be simplified as:

$$2\eta a_{n\times 1} = \frac{1}{2}T_{n\times n}\lambda_{n\times 1} + \mu_{n\times 1}. \tag{11}$$

It is useful to define two additional matrices. We define a lower triangular $(n\times n)$ matrix M,

$$M_{n\times n} = \begin{pmatrix} -1 & 0 & \cdots & 0 \\ -1 & -1 & \cdots & 0 \\ \vdots & \ddots & \ddots & \vdots \\ -1 & -1 & \cdots & -1 \end{pmatrix},$$

and a $(n \times (n+1))$ matrix L similar to matrix M, but with an extra column of zeros at the end, as follows:

$$L_{n\times(n+1)} = \begin{pmatrix} -1 & 0 & \cdots & 0 & 0 \\ -1 & -1 & \cdots & 0 & 0 \\ \vdots & \ddots & \ddots & \vdots & \vdots \\ -1 & -1 & \cdots & -1 & 0 \end{pmatrix}.$$

Both matrices can be expressed as:

$$M_{ij} = L_{ij} = \begin{cases} -1, & i >= j, \\ 0, & \text{otherwise}, \end{cases} \tag{12}$$

where $i = 1, \cdots, n$ for both matrices, but $j = 1, \cdots, n$ for M while $j = 1, \cdots, n+1$ for L. Using these definitions it is obvious that:

$$D_{n\times n} \cdot M_{n\times n} = I_{n\times n}.$$

which is the $(n \times n)$ identity matrix. Similarly it is easily to show that:

$$E_{(n+1)\times n} \cdot L_{n\times(n+1)} = J_{(n+1)\times(n+1)};$$

where matrix J is defined as:

$$J_{ij} = \begin{cases} 1, & i = j \quad \text{and} \quad i \neq n+1, \\ -1, & i = n+1 \quad \text{and} \quad j \neq n+1, \\ 0, & \text{otherwise}. \end{cases} \tag{13}$$

Since $DM = I$, we see that:

$$DM \times T\lambda = I \times T\lambda = T\lambda.$$

But from Eq. (10) note that $D\mu = T\lambda$, and therefore $DMT\lambda = D\mu$, it follows that:

$$\mu_{n\times 1} = M_{n\times n} T_{n\times n} \lambda_{n\times 1}.$$

We have defined above the matrix J as $EL = J$ and by multiplying the identity by $K(P-p)$ we find:

$$EL \times K(P-p) = J \times K(P-p),$$

Since $P-p=(P_0-p_0,P_1-p_1,\cdots,P_n-p_n)^T$ it follows that:

$$K_{(n+1)\times(n+1)}(P-p)_{(n+1)\times 1}=\begin{pmatrix}\sigma_0^{-2} & 0 & \cdots & 0\\ 0 & \sigma_1^{-2} & \cdots & 0\\ \vdots & \ddots & \ddots & \vdots\\ 0 & 0 & \cdots & \sigma_n^{-2}\end{pmatrix}\cdot\begin{pmatrix}P_0-p_0\\ P_1-p_1\\ \vdots\\ P_n-p_n\end{pmatrix}.$$

Which results in:

$$K(P-p)=\begin{pmatrix}\sigma_0^{-2}(P_0-p_0)\\ \sigma_1^{-2}(P_1-p_1)\\ \vdots\\ \sigma_n^{-2}(P_n-p_n)\end{pmatrix},$$

therefore;

$$\begin{aligned}J_{(n+1)\times(n+1)}(K(P-p))_{(n+1)\times 1} &= \begin{pmatrix}1 & 0 & 0 & \cdots & 0\\ 0 & 1 & 0 & \cdots & 0\\ \vdots & \ddots & \ddots & \ddots & \vdots\\ 0 & \cdots & \cdots & 1 & 0\\ -1 & -1 & \cdots & -1 & 0\end{pmatrix}\cdot\begin{pmatrix}\sigma_0^{-2}(P_0-p_0)\\ \sigma_1^{-2}(P_1-p_1)\\ \vdots\\ \sigma_n^{-2}(P_n-p_n)\end{pmatrix}\\ &= \begin{pmatrix}\sigma_0^{-2}(P_0-p_0)\\ \vdots\\ \sigma_{n-1}^{-2}(P_{n-1}-p_{n-1})\\ -\sum\limits_{i=1}^{n-1}\sigma_i^{-2}(P_i-p_i)\end{pmatrix}\\ &= E_{(n+1)\times n}L_{n\times(n+1)}(K(P-p))_{(n+1)\times 1}.\end{aligned}$$

Then using Eq. (9) it follows:

$$ELK(P-p)-E\lambda=\begin{pmatrix}\sigma_0^{-2}(P_0-p_0)\\ \vdots\\ \sigma_{n-1}^{-2}(P_{n-1}-p_{n-1})\\ -\sum\limits_{i=1}^{n-1}\sigma_i^{-2}(P_i-p_i)\end{pmatrix}-\begin{pmatrix}\sigma_0^{-2}(P_0-p_0)\\ \sigma_1^{-2}(P_1-p_1)\\ \vdots\\ \sigma_n^{-2}(P_n-p_n)\end{pmatrix}=\begin{pmatrix}0\\ \vdots\\ 0\\ -\sum\limits_{i=1}^{n}\sigma_i^{-2}(P_i-p_i)\end{pmatrix}.$$

Since $\sum\limits_{i=1}^{n}\sigma_i^{-2}(P_i-p_i)=0$ holds from Eq. (1), if we add the whole identities in this equation together, it follows that $ELK(P-p)=E\lambda$ and it is found:

$$\lambda_{n\times 1}=L_{n\times(n+1)}(K(P-p))_{(n+1)\times 1}.$$

Using the last two identities for μ and λ in Eq. (11) we get: $2\eta a = \frac{1}{2}TLK(P-p) + MTLK(P-p)$ which can be written as:

$$2\eta a = (\frac{1}{2}TL + MTL)K(P-p). \tag{14}$$

To combine this Eq. (14) with Eq. (7) we define a $(n-1)\times n$ matrix G as follow:

$$G_{(n-1)\times n} = \begin{pmatrix} T_0^2T_1 & T_0T_1^2 & 0 & \cdots & 0 \\ 0 & T_1^2T_2 & T_1T_2^2 & \cdots & 0 \\ \vdots & \ddots & \ddots & \ddots & \vdots \\ 0 & 0 & \cdots & T_{n-2}^2T_{n-1} & T_{n-2}T_{n-1}^2 \end{pmatrix},$$

which can be expressed as:

$$G_{ij} = \begin{cases} T_{i-1}^2T_i, & i=j, \\ T_{i-1}T_i^2, & i+1=j, \\ 0, & \text{otherwise.} \end{cases} \tag{15}$$

And a $(n-1)\times(n+1)$ matrix B such that:

$$B_{(n-1)\times(n+1)} = \begin{pmatrix} T_1 & -(T_0+T_1) & T_0 & 0 & \cdots & 0 \\ 0 & T_2 & -(T_1+T_2) & T_1 & \cdots & 0 \\ \vdots & \ddots & \ddots & \ddots & \ddots & \vdots \\ 0 & 0 & \cdots & T_{n-1} & -(T_{n-2}+T_{n-1}) & T_{n-2} \end{pmatrix},$$

which can be written for $i = 1, \cdots, (n-1)$ as:

$$B_{ij} = \begin{cases} T_i, & i=j, \\ -(T_{i-1}+T_i), & j=i+1, \\ T_{i-1}, & j=i+2, \\ 0, & \text{otherwise.} \end{cases} \tag{16}$$

Note that Eq. (7) is equivalent to:

$$p_{i+1}T_{i-1} - p_i(T_i + T_{i-1}) + p_{i-1}T_i = \frac{1}{2}(a_iT_{i-1}T_i^2 + a_{i-1}T_{i-1}^2T_i).$$

Using the definitions of matrices G and B it follows that Eq. (7) can be written as:

$$B_{(n-1)\times(n+1)}p_{(n+1)\times 1} = \frac{1}{2}G_{(n-1)\times n}a_{n\times 1}, \tag{17}$$

and from Eq. (14) we see that $a = \frac{1}{2\eta}(\frac{1}{2}TL + MTL)K(P - p)$. Substituting a in Eq. (7) we get:

$$\eta Bp = \frac{1}{4}G(\frac{1}{2}TL + MTL)K(P - p).$$

We define a $(n-1) \times (n+1)$ matrix A as:

$$A_{(n-1)\times(n+1)} = \frac{1}{4}G_{(n-1)\times n}(\frac{1}{2}TL + MTL)_{n\times(n+1)}, \tag{18}$$

and find $\eta Bp = AK(P - p)$. Therefore:

$$(AK + \eta B)p = AKP. \tag{19}$$

Finally to include the condition $(\sum_{i=1}^{n}\sigma_i^{-2}(P_i - p_i) = 0)$ we define a $n \times (n+1)$ matrix $\bar{B}$ to be matrix B augmented with a final row of zeros, and a $n \times (n+1)$ matrix $\bar{A}$ to be A augmented with a final row of ones, then we get the equation:

$$(\bar{A}K + \eta\bar{B})p = \bar{A}KP. \tag{20}$$

We could solve this system of equations using the eigenvalues and eigenvectors of these matrices, but in practical applications the dimensions of these matrices are quite large. Therefore we use the singular value decomposition method to obtain the least squares approximate solution p for the observed positions P for a given parameter η. Because matrices A and B have an essentially lower triangular form, the approximation errors are largest for the p_i with largest i, which is not what wanted for state estimation and tracking, our desire is to have the smallest errors at the most recent times. We solve this problem just by reversing the columns and the rows of matrices $\bar{A}$ and $\bar{B}$ to obtain the desired solution[1].

[1] See (Judd, 2015) section II-C
